# Long Non-Coding RNA-TMPO-AS1 as ceRNA Binding to let-7c-5p Upregulates STRIP2 Expression and Predicts Poor Prognosis in Lung Adenocarcinoma

**DOI:** 10.3389/fonc.2022.921200

**Published:** 2022-06-14

**Authors:** Juan Wang, Yixiao Yuan, Lin Tang, Haoqing Zhai, Dahang Zhang, Lincan Duan, Xiulin Jiang, Chen Li

**Affiliations:** ^1^ Department of Thoracic Surgery, The Third Affiliated Hospital of Kunming Medical University, Kunming, China; ^2^ Laboratory of Animal Models and Human Disease Mechanisms of Chinese Academy of Sciences & Yunnan Province, Kunming Institute of Zoology, Kunming, China; ^3^ Department of Biology, Chemistry, Pharmacy, Free University of Berlin, Berlin, Germany

**Keywords:** striatin-interacting protein 2, prognosis biomarkers, tumor immune infiltration, lung adenocarcinoma, cell proliferation

## Abstract

**Background:**

Striatin-interacting protein 2 (STRIP2), also called Fam40b, has been reported to regulate tumor cell growth. But the role of STRIP2 in lung adenocarcinoma (LUAD) has not been discovered clearly. Thus, the aim of our study is to explore the function and underlying mechanism of STRIP2 in LUAD.

**Methods:**

Expression of STRIP2 was determined using the Cancer Genome Atlas (TCGA), GTEx, Ualcan, and the Human Protein Altas databases. The Correlation of STRIP2 and survival was detected by PrognoScan and Kaplan–Meier plotter databases. Besides, the correlation between STRIP2 expression and tumor immune infiltration as well as immune checkpoints were analyzed by the ssGSEA method. The biological function of STRIP2 and its co-expression genes was determined by gene ontology (GO) and Genes and Genomes (KEGG), respectively. Finally, the expression level and biological function of STRIP2 in LUAD were determined by qPCR, CCK8, transwell, and wound healing assays.

**Results:**

This manuscript revealed a significantly increased expression of mRNA and protein of STRIP2 in lung adenocarcinoma compared with the adjacent normal tissues. GEO and Kaplan–Meier plotter databases showed higher STRIP2 expression levels were correlated with poor prognosis survival of LUAD. Moreover, Cox regression analysis suggested that a higher STRIP2 level served as an independent risk factor in predicting deteriorative overall survival (OS) for LUAD patients. SsGSEA results showed STRIP2 expression level was positively correlated with infiltrating levels of Th2 cells in LUAD. Lastly, GO analysis indicated the biological processes were enriched in nuclear division and positive regulation of the cell cycle. KEGG signaling pathway analysis showed STRIP2 was correlated with the MAPK signaling pathway and the TNF signaling pathway. The GSEA database showed that STRIP2 was positively associated with the epithelial–mesenchymal transition, cell cycle, and TNF signaling pathway. The QRT-PCR assay showed that STRIP2 was upregulated in LUAD cell lines. Cell proliferation and migration were inhibited in LUAD by knockdown of STRIP2. Moreover, we confirmed that the TMPO-AS1/let-7c-5p/STRIP2 network regulates STRIP2 overexpression in LUAD and is associated with poor prognosis.

**Conclusion:**

Our findings indicated that STRIP2 acted as a crucial oncogene in LUAD and was correlated with unfavorable survival and tumor infiltration inflation.

## Introduction

Lung cancer is a worldwide epidemic malignancy among both women and men, and terribly causes globally cancer-related deaths (1). In China, the incidence and deaths of lung cancer have increased rapidly in recent years, revealing geographic and gender differences (2). Lung adenocarcinoma (LUAD) accounts for approximately more than 40% of the lung cancer incidence and poses a systemic threat due to more frequently occurring distant metastasis in LUAD (3). LUAD, with an overall survival of less than 5 years, is the most aggressive and rapid metastasis (4). For the past few years, immunotherapy has been highlighted as very effective in lung cancer therapy; however, it also has previously failed in lung cancer (5, 6). Therefore, the terrible therapeutic results of immunotherapy are the new obstacles for clinical application of immune checkpoint inhibitors in LUAD.

STRIP2 is a member of the striatin-interacting phosphatase and kinase (STRIPAK) complex that participates in regulating cell growth and migration (7). Increased STRIP2 has been observed in many cancers and is correlated with poor prognosis and unfavorable clinicopathological characteristics in gastric cancer and breast cancer cells (8, 9). High expression of STRIP2 implicates neoplastic growth, metastasis, chemoresistance, and shorter survival time in various human cancers (9). Increasing evidence has indicated that STRIP2 exerts its role as an oncogene by regulating various signaling pathways to regulate tumorigenesis and progression and counteract the effects of many chemotherapies. For instance, STRIP2 is involved in the P38-AKT-MMP-2 signaling pathway to regulate mouse aortic smooth muscle cell (MOVAS) proliferation and migration (10). There is little but strong evidence to demonstrate the connection between STRIP2 and immune cell infiltration in cancer. Despite STRIP2 serving as an oncogene in LUAD, no evidence demonstrates the association between STRIP2 and immune cell infiltration in LUAD.

Therefore, in this study, we detected the connection between STRIP2 and prognosis and immune infiltration in tumor samples of LUAD patients. Furthermore, GSEA and KEGG enrichment analyses were conducted to examine the potential signaling pathway of STRIP2 in LUAD progression. Finally, we performed a loss of function assay to determine the biological function of STRIP2 in LUAD. Our data highlight the crucial role of STRIP2 in tumor initiation, progression, clinical outcome, and immune infiltration in LUAD.

## Materials and Methods

### TCGA Datasets

We downloaded the RNA expression data and corresponding clinical information from the TCGA official website (https://portal.gdc.cancer.gov/). We used this data analysis to examine the correlation between STRIP2 expression and relevant clinical information, including pathological stage, andTNM stage.

### UALCAN Database

UALCAN (http://ualcan.path.uab.edu/) is an online resource for the analysis of TCGA gene expression data (11). In this finding, we used UALCAN to examine the protein level of STRIP2 in LUAD.

### The Human Protein Atlas (HPA)

HPA (https://proteinatlas.org/ contains information on normal tissue and tumor tissue protein levels of human gene expression profiles (12). In this study, we explored the protein expression of STRIP2 in lung cancer tissue.

### The Clinical Proteomic Tumor Analysis Consortium (CPTAC) Common Data Analysis

The Clinical Proteomic Tumor Analysis Consortium (CPTAC) has produced large proteomics data sets from the mass spectrometric interrogation of tumor samples previously analyzed by The Cancer Genome Atlas (TCGA) program (13). In this study, we determined the expression of STRIP2 in lung cancer using the Clinical Proteomic Tumor Analysis Consortium (CPTAC) data.

### AnnoLnc2 Database

AnnoLnc2 (http://annolnc.gao-lab.org/) is a one-stop portal to systematically annotate novel lncRNAs for humans and mice. lncRNAs with a comprehensive functional spectrum covering sequences, structure, expression, regulation, genetic association, and evolution (14). In this study, we used the AnnoLnc2 database to examine the subcellular localization and molecular coding potential of lncRNAs.

### Gene Set Enrichment Analysis

In this study, we used the linkedomics database (http://www.linkedomics.org/login.php) to obtain the co-expression genes of STRIP2 in LUAD. We used the GSEA software and clusterProfiler package to perform KEGG enrichment analysis of the signaling pathway of STRIP2 in LUAD (15–17).

### Cell Culture

The BEAS-2B cell line was purchased from the Cell Bank of theKunming Institute of Zoology and was cultured in BEGM media (Lonza, CC-3170). Lung cancer cell lines, namely, h1650, A549, SPC-A1, and H1975, were purchased from Cobioer, China with STR documents, and were cultured in RPMI-1640 medium (Corning) supplemented with 10% fetal bovine serum (FBS) and 1% penicillin/streptomycin.

### Cell Proliferation and Cell Migration Assay

The cell proliferation assay was performed as previously described (18). Indicated tumor cells were plated onto 12-well plates. The cell numbers were subsequently counted each day using an automatic cell analyzer countstar (Shanghai Ruiyu Biotech Co.). For the trans-well migration assay, 2 × 10^4^ cells/well in 100 μl serum-free medium were plated in a 24-well plate chamber insert, and the lower chamber was filled with 10% FBS. After incubation for 24 h, cells were fixed with 4% PFA, washed and then stained with 0.5% crystal violet for further pictures to be captured.

### Real-Time RT-PCR Assay

Using a real-time RT-PCR assay, cells were lysed by RNAiso Plus (Takara Bio, Beijing, China, Cat. 108-95-2). The primer used in this study is as follows: β-actin-F: AAGTGTGACGTGGACATCCGC, β-actin-R: CCGGACTCGTCATACTCCTGCT, STRIP2-F: AGGTGGTCAGTAGGGAACGG, and STRIP2-R: TGTAGCACATCGACCTCTGAA.

### Statistical Analyses

**Figure 1:**
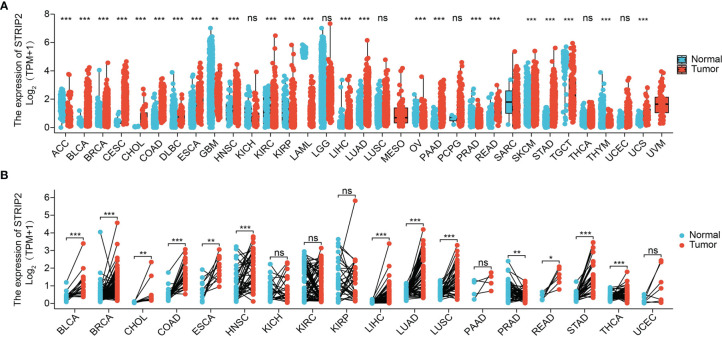
Expression pattern of STRIP2 from the perspective of pan-cancer. **(A)** STRIP2 was highly expressed in 24 of the 33 cancers compared with normal tissue. **(B)** The expression of STRIP2 in paired cancer tissues and adjacent normal tissues from the TCGA datasets. Cancer full name in **Table 2**. NS: P >0.05,*P <0.05, **P <0.01, ***P <0.001.

The statistical analyses for **Figures 1** **–** **4** were performed using R (V 3.6.3) and ROC curves to detect STRIP2 cutoff values using pROC packages. GraphPad Prism 7.0 was used for statistical analyses of the data regarding the biological function of STRIP2 (**Figure 9**). The significance of the data between two experimental groups was determined by Student’s t-test, and multiple group comparisons were analyzed by one-way ANOVA. P <0.05 (*), P <0.01 (**), and P <0.001 (***) were significant.

**Figure 2:**
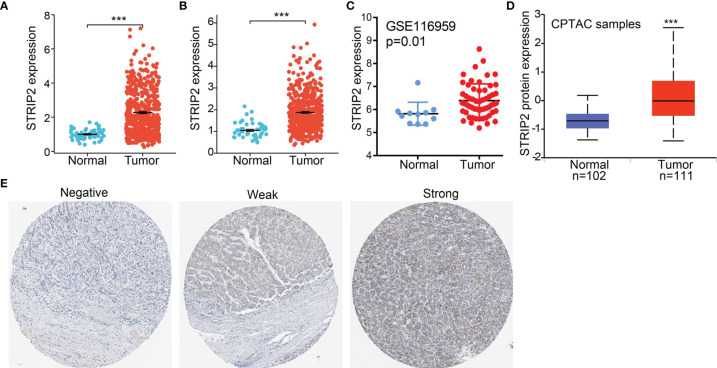
STRIP2 RNA and protein expression in LUAD. **(A, B)** STRIP2 mRNA expression levels in LUAD and LUSC patients and matched adjacent normal samples. **(C)** Validate the expression of STRIP2 in lung cancer. **(D)** STRIP2 protein expression level based on CPTAC. **(E)** STRIP2 protein levels based on Human Protein Atlas. ***P <0.001.

**Figure 3:**
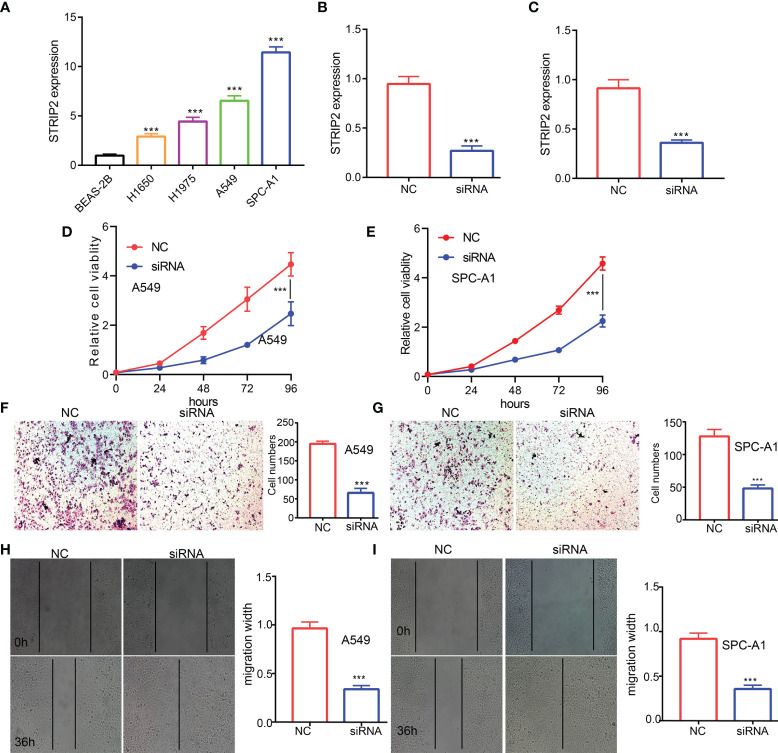
STRIP2 regulates LUAD cell proliferation and migration. **(A)** qPCR assay examines the expression level of STRIP2 in lung adenocarcinoma cancerous cell lines, namely, H1975, A549, and SPC-A1, compared to normal human bronchial epithelial cell line: BEAS-2B. **(B, C)** Establishment of STRIP2 knockdown cell lines in A549 and SPC-A1 verified by Real-time RT-PCR **(D-I)** Knockdown of STRIP2 significantly inhibits cell proliferation and migration in H1975 cells, as measured by CCK8, transwell, and Wound healing assays. NC, negative control; siRNA, STRIP2 siRNA. ***p <0.001.

**Figure 4:**
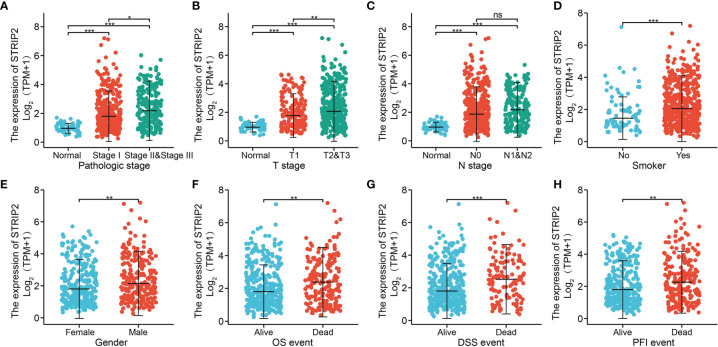
Clinical significance of STRIP2 in lung adenocarcinoma. Correlation between STRIP2 expression and clinical parameters, including, **(A)** pathological stage, **(B–D)** TNM stage, **(E)** gender, **(F–H)** OS, DSS, and PFS events. NS: P >0.05, *P <0.05, **P <0.01, ***P <0.001.

## Results

### Expression Level of STRIP2 in Human Cancers

STRIP2 expression was explored across tumor types in the TCGA database and GETx, followed by paired-difference analysis. Results show that STRIP2 was increased in 19 of the 33 cancers compared with normal tissue **(** **Figure 1A** **)**. We also found that STRIP2 expression in paired cancer tissues and adjacent normal tissues in pan-cancer employed TCGA datasets. We found that STRIP2 levels were significantly higher in 12 of the 18 cancers compared with normal tissue **(** **Figure 1B** **)**.

### STRIP2 Was Upregulated in Lung Adenocarcinoma

To further determine STRIP2 mRNA and protein expression in LUAD, we analyzed STRIP2 expression data in TCGA and HPA. We found that STRIP2 was more highly expressed in LUAD and lung squamous cell carcinoma (LUSC) tumor tissues than in normal lung tissue **(** **Figures 2A, B** **)**. Consistent with the results from the TCGA data, STRIP2 was significantly increased in lung tissue based on the Gene Expression Omnibus (GEO) dataset **(** **Figure 2C** **)**. Moreover, we found that STRIP2 protein expression in LUAD was significantly higher than that in normal tissue **(** **Figure 2D** **)**. As shown in **Figure 2D**, immunohistochemistry (IHC) results also confirmed that the upregulation of STRIP2 protein expression in lung cancer tissue compared to non-cancerous tissue **(** **Figure 2E** **)**.

### Depletion of *STRIP2* Significantly Suppressed Proliferation and Migration of LUAD Cells

To examine the expression of *STRIP2*, we detected *STRIP2* expression levels in LUAD cell lines using a qRT-PCR assay. Results confirmed that *STRIP2* was significantly increased in lung cancer cell lines, especially in A549 and H1975 cells (**Figure 3A**). The qRT-PCR assay showed that the expression of *STRIP2* mRNA was significantly decreased in H1975 cells after treatment with targeted siRNA (**Figures 3B, C**). The cholecystokinin octapeptide (CCK8), transwell, and wound healing assays demonstrated that STRIP2 depletion significantly inhibits the cell proliferation and cell migration ability of LUAD (**Figures 3D–I**). Collectively, these results demonstrate that *STRIP2* was highly expressed in LUAD and significantly affected their proliferation and cell cycle.

### Correlation Between STRIP2 Expression and Clinical Parameters

To examine the relationship between STRIP2 expression and clinical–pathological features in a LUAD sample. We found that STRIP2 expression was significantly associated with pathological stage, TN stage, smoking, gender, OS event, DSS event, and PFS event in patients with LUAD **(** **Figures 4A–H).** Moreover, logistics analysis results also show that upregulation of STRIP2 correlated with T stage (T2 & T3 & T4 *vs.* T1), N stage (N2 *vs.* N1), pathologic stage (Stage III & Stage IV *vs.* Stage I & Stage II), Gender (Male *vs.* Female), and smoker (Yes *vs.* No) **(** **Table 1** **)**.

**Table 1:** Logistic regression analyzed the correlation between STRIP2 expression and clinical–pathological characteristics in LUAD.

Characteristics	Total (N)	Odds Ratio (OR)	P–value
T stage (T2 & T3 & T4 *vs.* T1)	532	1.606 (1.117–2.319)	0.011
N stage (N2 *vs.* N1)	169	1.265 (0.685–2.350)	0.453
Pathologic stage (Stage III & Stage IV *vs.* Stage I & Stage II)	527	1.723 (1.127–2.659)	0.013
Gender (Male *vs.* Female)	535	1.635 (1.162–2.305)	0.005
Smoker (Yes *vs.* No)	521	2.248 (1.356–3.811)	0.002

**Table 2:** Cancer type and full name in TCGA database.

Tumor	Full name	Tumor	Full name
ACC	Adrenocortical carcinoma	LGG	Brain Lower Grade Glioma
BLCA	Bladder Urothelial Carcinoma	LIHC	Liver hepatocellular carcinoma
BRCA	Breast invasive carcinoma	LUAD	Lung adenocarcinoma
CESC	Cervical squamous cell carcinoma and endocervical adenocarcinoma	LUSC	Lung squamous cell carcinoma
CHOL	Cholangiocarcinoma	MESO	Mesothelioma
COAD	Colon adenocarcinoma	OV	Ovarian serous cystadenocarcinoma
DLBC	Lymphoid Neoplasm Diffuse Large B-cell Lymphoma	PAAD	Pancreatic adenocarcinoma
ESCA	Esophageal carcinoma	PCPG	Pheochromocytoma and Paraganglioma
GBM	Glioblastoma multiforme	PRAD	Prostate adenocarcinoma
HNSC	Head and Neck squamous cell carcinoma	READ	Rectum adenocarcinoma
KICH	Kidney Chromophobe	SARC	Sarcoma
KIRC	Kidney renal clear cell carcinoma	SKCM	Skin Cutaneous Melanoma
KIRP	Kidney renal papillary cell carcinoma	STAD	Stomach adenocarcinoma
LAML	Acute Myeloid Leukemia	TGCT	Testicular Germ Cell Tumors
THCA	Thyroid carcinoma	THYM	Thymoma
UCEC	Uterine Corpus Endometrial Carcinoma	UVM	Uveal Melanoma
UCS	Uterine Carcinosarcoma		

### STRIP2 Was Upregulated in LUAD and Predicts an Unfavorable Prognosis for LUAD Patients

**Figure 5:**
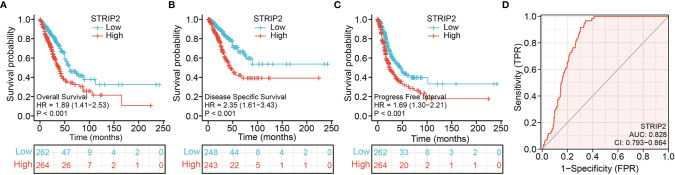
ROC and Kaplan–Meier curves of STRIP2. **(A–C)** Kaplan–Meier survival curves showed that lung adenocarcinoma patients with high STRIP2 expression exhibited poor overall survival, disease-specific survival, and progression-free survival of STRIP2 in LUAD determine by the TCGA-LUAD dataset. **(D)** ROC curves were used to determine the diagnostic value of STRIP2 in lung adenocarcinoma.

**Figure 6:**
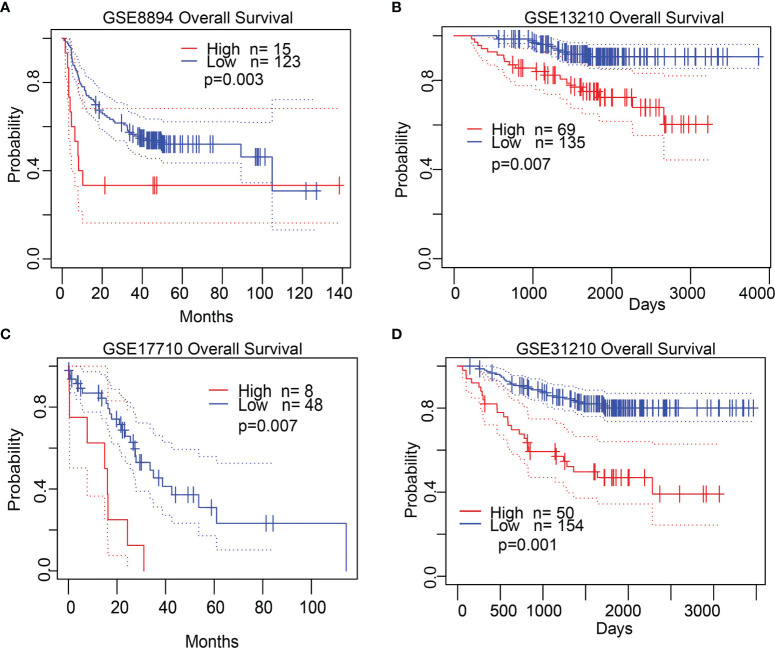
Validate the prognostic value of STRIP2 in lung cancer. **(A**–**D)** Kaplan–Meier survival curves showed that lung adenocarcinoma patients with high STRIP2 expression exhibited poor overall survival determine by the GEO dataset.

Next, we analyzed the expression and clinical significance of STRIP2 in the TCGA database. We found that colorectal cancer (CRC) patients with higher STRIP2 levels had shorter overall survival, disease-specific survival, and progression survival **(** **Figures 5A–C** **)**. We further examined the diagnostic value of STRIP2 in distinguishing LUAD samples from normal lung tissue. Receiver operating characteristic (ROC) curve analysis confirmed that the AUC value of STRIP2 was 0.828, with a 95% CI = 0.793–0.864 **(** **Figure 5D** **)**. Then, we determined the prognostic values of STRIP2 in 4 independent GEO cohorts of lung cancer samples. Consistent with the results from the TCGA data, Kaplan–Meier analysis showed that patients with higher STRIP2 expression were associated with reduced overall survival time **(** **Figures 6A–D** **)**. Collectively, these results suggest that STRIP2 was upregulated in lung cancer and that high expression levels of STRIP2 were associated with poor outcomes in lung cancer patients.

### Validation of the Prognostic Value of STRIP2 Based on Various Subgroups

**Figure 7:**
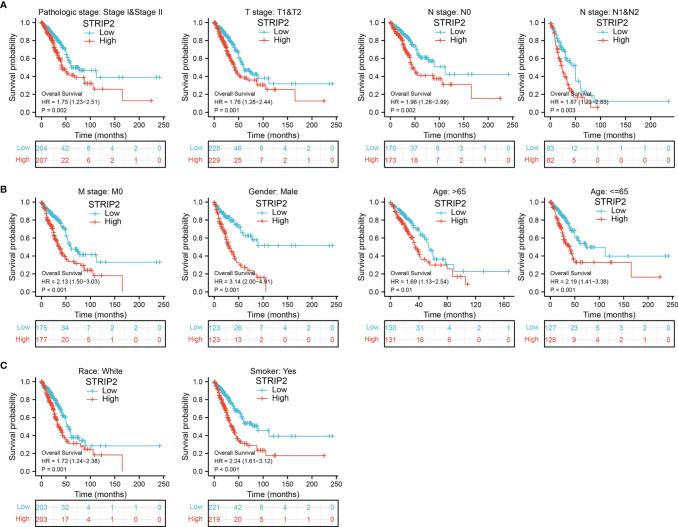
The overall survival of STRIP2 based on diverse subgroup. **(A**–**C)** Correlations between STRIP2 expression level and the overall survival in different clinical subgroups of LUAD in the TCGA database, including, stage I and II, T1 and T2, N0 and N1, M0, CR, Smoker, Gender, Age, and white.

We further determined the prognostic values of STRIP2 in various clinical subgroups, namely, the pathological stage, tumor-node-metastasis (TNM) stage, gender, age, race, and smokers. Results suggest that upregulated STRIP2 levels are associated with poor clinical outcomes in patients with lung cancer **(** **Figures 7A–C).**

### Univariate and Multivariate Cox Regression Analyses of Different Parameters on Overall Survival

**Figure 8:**
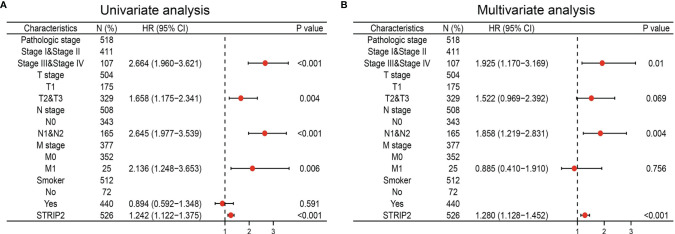
Forest plot of univariate and multivariate Cox regression analysis in LUAD. **(A, B)** The forest plot of univariate and multivariate Cox regression analysis in LUAD.

We performed Univariate Cox regression analysis in the TCGA-LAUD cohort to determine whether STRIP2 expression level might be a valuable prognostic biomarker. Univariate Cox regression analysis results show that high expression of STRIP2, pathologic stage, and TNM stage were associated with overall survival in LUAD patients **(** **Figure 8A** **)**. Multivariate Cox regression analysis was performed to ascertain whether STRIP2 expression level could be an independent prognostic factor for patients with LUAD. We confirmed that increased STRIP2 expression was a significant independent prognostic factor in the TCGA-LAUD cohort that directly correlated with adverse clinical outcomes, along with pathological stage and N stage **(** **Figure 8B** **)**.

### Construction and Validation of STRIP2 Based Nomogram

**Figure 9:**
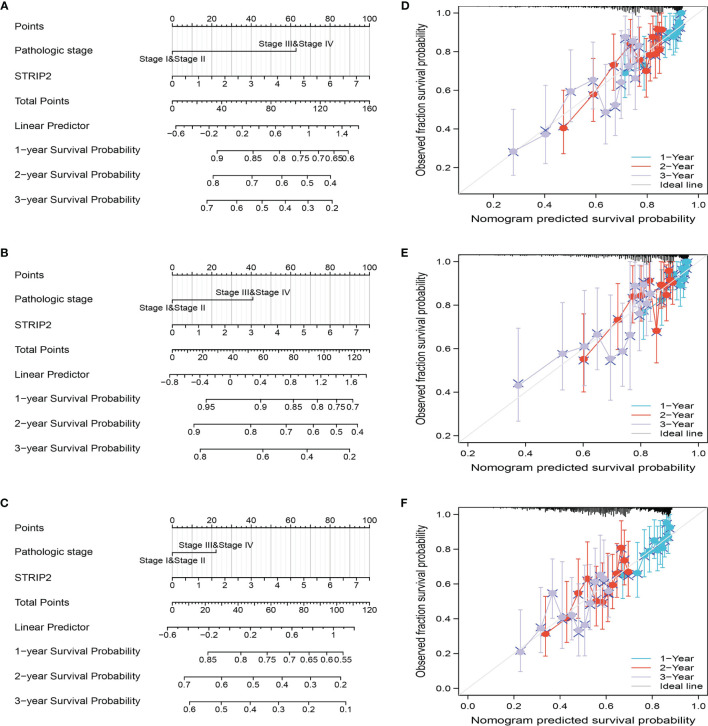
Construction and performance validation of the STRIP2 based nomogram for lung adenocarcinoma patients. **(A**–**F)** The calibration curve and Hosmer–Lemeshow test of nomograms in the TCGA-lung adenocarcinoma cohort for overall survival, disease-specific survival and progression-free survival.

The results of the multivariate analysis confirmed that STRIP2 is an independent prognostic factor in LUAD. We then constructed a prediction model for overall survival, disease-free survival, and progression-free survival by integrating STRIP2 expression and pathological stage. We established a nomogram to integrate STRIP2 as a LUAD biomarker. Higher total points on the nomogram for overall survival, progression-free interval (PFI), and disease-specific survival, respectively, indicated a worse prognosis **(** **Figures 9A–F** **).**

### KEGG and GSEA Enrichment Analysis

**Figure 10:**
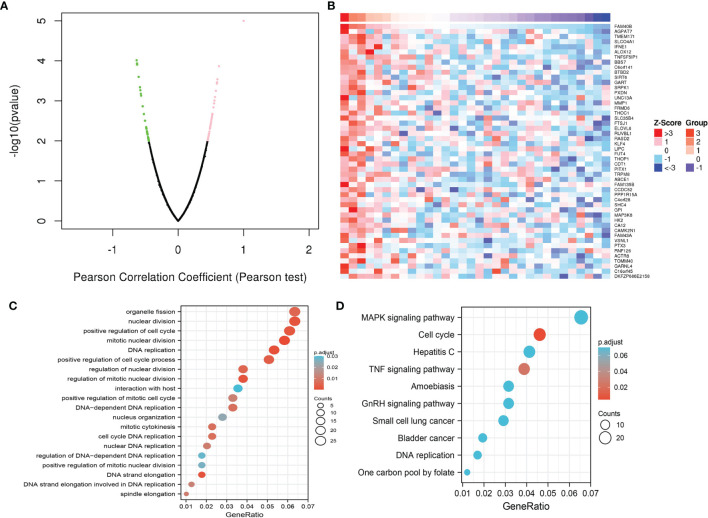
Functional enrichment analysis. **(A, B)** The correlation analysis of STRIP2 expression and its top 100 co-expressed gene network. **(C, D)** GO and KEGG enrichment analysis of co-expressed genes.

As shown in **Figures 10A, B**, the Linkedomic database is used to obtain the top 100 co-expressed genes by Pearson’s correlation analysis. In terms of biological processes, STRIP2 is mainly involved in organelle fission, nuclear division, positive regulation of the cell cycle, DNA replication, positive regulation of the cell cycle process, regulation of nuclear division, and positive regulation of the mitotic cell cycle **(** **Figure 10C** **)**. KEGG enrichment analysis suggested that these genes participated in the MAPK signaling pathway, cell cycle, TNF signaling pathway, GnRH signaling pathway, and DNA replication **(** **Figure 10D** **)**.

**Figure 11:**
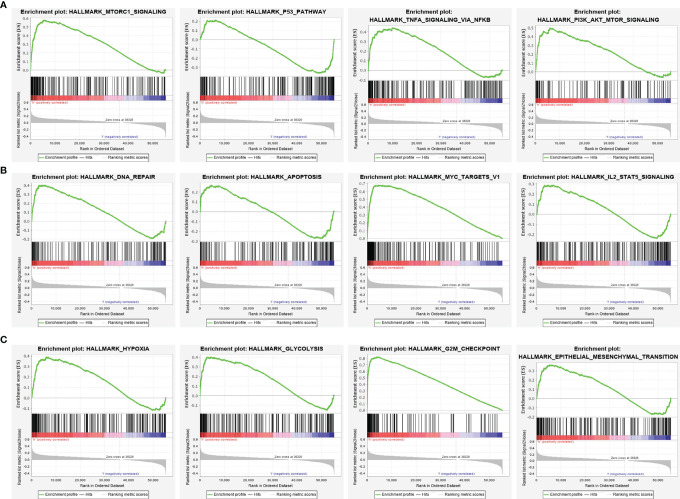
Identification of STRIP2 related signaling pathways in lung adenocarcinoma. **(A**–**C)** Identification of STRIP2 related signaling pathways by GSEA software.

To explore the possible mechanism of *STRIP2* in LUAD, GSEA analysis was performed on the differential genes. Gene set enrichment analysis (GSEA) also showed that pathways, namely, the MTOR signaling pathway, P53 signaling pathway, PI3K AKT MTOR signaling pathway, TNFA signaling pathway, DNA replication, apoptosis, IL2 STAT5 signaling pathway, MYC targets, hypoxia, GLYCOSIS, G2M Checkpoint, and EMT were significantly enriched in the high *STRIP2* expression group **(** **Figures 11A–C** **)**.

### Correlation Between STRIP2 Expression and Immune Infiltration

**Figure 12:**
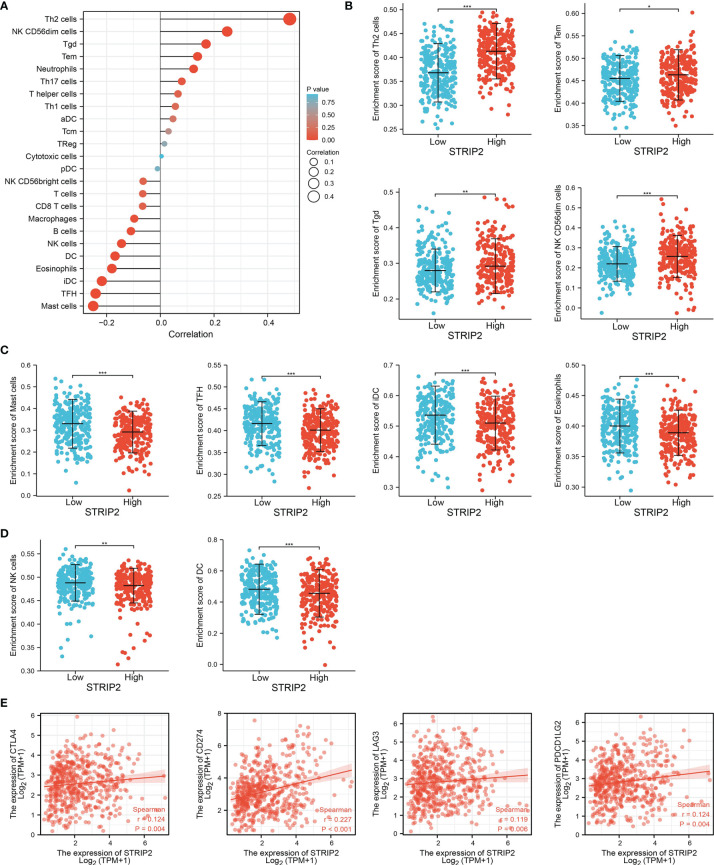
Correlation analysis of STRIP2 expression and infiltration levels of immune cells in LUAD. **(A**–**D)** The correlation between STRIP2 expression and the infiltration levels of 24 immune cells in LUAD by ssGSEA. **(E)** Correlation between STRIP2 and immune check points genes in LUAD. NS: P >0.05,*P <0.05, **P <0.01, ***P <0.001.

Given that the gene set enrichment analysis (GSEA) enrichment analysis above showed that STRIP2 may be correlated with the immune response regulation, we therefore examined the association between *STRIP2* expression levels and immune cell infiltration, we used the ssGSEA algorithm to quantify the level of immune cell infiltration in the high- and low-expression groups of STRIP2. We determined that increased expression of *STRIP2* was positively associated with the abundance of Th2 cells, NK CD56dim cells, Tgd, Tem, and neutrophils, and negatively associated with the abundance of macrophages, B cells, NK cells, DC, eosinophils, iDC, TFH, and mast cells in LUAD **(** **Figures 12A–D** **)**.

Given that immune checkpoints play a crucial role in tumor immunosuppression, we analyzed the correlation between STRIP2 expression and that of the immune checkpoint-related genes in LUAD using Pearson’s correlation analysis. The results confirmed that STRIP2 expression was significantly positively correlated with the expression of CD274, CTLA4, LAG3, and PDCD1LG2 in LUAD **(** **Figure 12E** **)**. These results confirm that STRIP2 plays a crucial role in immune infiltration in LUAD.

### Construction of the lncRNA–miRNA–mRNA Triple Regulatory Networks

**Figure 13:**
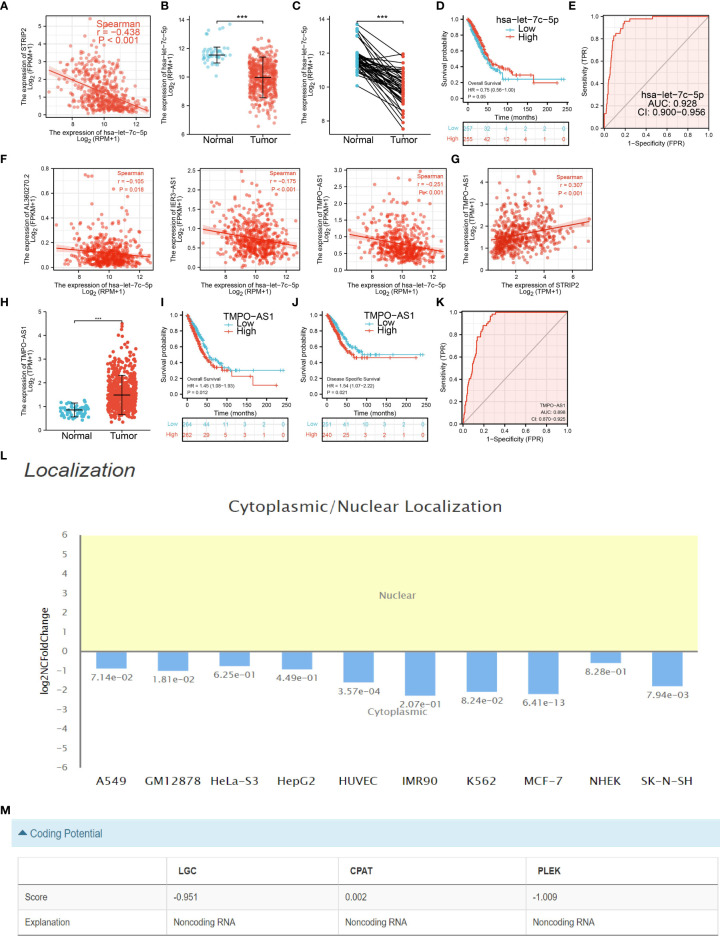
Analysis of the potential miRNAs and lncRNAs of STRIP2. **(A)** Correlations between STRIP2 expression and has-let-7c-5p in LUAD. **(B**–**D)** The expression level and prognostic value of has-let-7c-5p in LUAD. **(E)** ROC curve analyses and AUC values for has-let-7c-5p in lung cancer patients. **(F)** Correlations between has-let-7c-5p expression and lncRNAs (TMPO-AS1, IER3-AS1, and AL360270.2) in LUAD. **(G)** Correlations between STRIP2 expression and lncRNA-TMPO-AS1 in LUAD. **(H**–**K)** The expression levels, prognostic and diagnostic values of lncRNA-TMPO-AS1. **(L, M)** Subcellular localization and coding potential of lncRNA-TMPO-AS1 in LUAD, ***P <0.001.

To examine the lncRNA–miRNA–mRNA networks that modulate the expression of STRIP2 in LUAD, the Starbase database employed to obtain the potential miRNAs that bind with STRIP2, we obtained the 45 STRIP2-related miRNAs to construct a miRNA–mRNA co-expression network **(** **Supplementary Table 1** **)**. In the miRNA–mRNA correlation analysis, we found a correlation between let-7c-5p expression and STRIP2 expression in LUAD (cor<−0.438, P-value <0.001; **Figure 13A** **)**, and we found that let-7c-5p expression was significantly reduced in LUAD than in normal tissues and correlated with poor clinical outcomes **(** **Figures 13B, C** **)**. ROC curve analysis of let-7c-5p showed an AUC value of 0.928 in lung cancer patients **(** **Figure 13D** **).** We also used the Starbase database to identify 62 potential target lncRNAs correlated with let-7c-5p in LUAD **(** **Supplementary Table 2** and **Figure 13E** **)**. Among them, TMPO-AS1, IER3-AS1, and AL360270.2 met the filtering conditions for correlation analysis with let-7c-5p (cor<−0.1, P-value <0.01), and only TMPO-AS1 was positively correlated with STRIP2 (cor >0.3, P-value <0.001) **(** **Figures 13F, G** **).** We also found that only TMPO-AS1 was significantly up-regulated in LUAD than in normal tissues, and its higher expression was associated with adverse clinical outcomes in patients with LUAD **(** **Figures 13H–J** **)**. ROC curve analysis of TMPO-AS1 showed an AUC value of 0.898 in lung cancer patients **(** **Figure 13K** **).** Cellular localization analysis confirmed that TMPO-AS1 had the highest percentage in the cytoplasm and could not be translated into coding-proteins **(** **Figures 13L, M** **)**. Since STRIP2 is upregulated in LUAD and associated with a poor prognosis, the TMPO-AS1/let-7c-5p/STRIP2 regulatory network could be used as a biomarker for poor prognosis and a new target for treating LUAD.

## Discussion

In this study, we found that STRIP2 expression was significantly higher in the LUAD than in the normal tissue at both transcriptional and protein levels. It was also upregulated in LUAD cell lines. The Kaplan–Meier curves and univariate analysis confirmed that *STRIP2* expression is correlated with overall survival (OS), disease-specific survival (DSS), and progression-free survival (PFS) in the LUAD patients of the TCGA data. The GEO dataset also confirmed that patients with a high level of STRIP2 correlated with adverse clinical outcomes. Moreover, Cox regression analysis suggested that a higher STRIP2 level served as an independent risk factor in predicting deteriorative OS for LUAD patients. ROC curve analysis indicated that *STRIP2* is a promising diagnostic biomarker for differentiating LUAD from normal tissues. We also established a nomogram to integrate *STRIP2* as a LUAD biomarker; higher total points on the nomogram for overall survival, progression-free interval (PFS), and disease-specific survival, respectively, indicated a worse prognosis.

Previous studies have reported that STRIP2 is necessary for the onset of embryonic stem cell differentiation (19). Morpholino-mediated knockdown of STRIP2 results in severe abnormalities of the cardiovascular system (20). To further examine the functional role of STRIP2 in LUAD, we conducted an enrichment analysis between high- and low-expression groups based on STRIP2 mRNA expression. KEGG enrichment analysis suggested that these genes participated in the MAPK signaling pathway, cell cycle, TNF signaling pathway, GnRH signaling pathway, and DNA replication. Likewise, results of the GSEA analysis revealed that upregulated STRIP2 expression was associated with MTOR signaling pathway, P53 signaling pathway, PI3K AKT the MTOR signaling pathway, TNFA signaling pathway, DNA replication, apoptosis, IL2 STAT5 signaling pathway, MYC targets, hypoxia, GLYCOSIS, G2M Checkpoint, and EMT.

A previous study found that STRIP2 expression predicts prognosis in gastric cancer (8). In this finding, we found that STRIP2 was positively associated with the abundance of Th2 cells, NK CD56dim cells, Tgd, Tem, and neutrophils, and negatively associated with the abundance of macrophages, B cells, NK cells, DC, eosinophils, iDC, TFH, and mast cells in LUAD. Moreover, STRIP2 expression was significantly positively correlated with the expression of CD274, CTLA4, LAG3, and PDCD1LG2 in LUAD. These results confirm that STRIP2 plays a crucial role in immune infiltration in LUAD.

Given that GSEA enrichment results shown that STRIP2 may plays a central role in cell proliferation and EMT. We decide to examine the potential biological function of STRIP2 in LUAD. *In vitro*, we found that STRIP2 was increased in LUAD cells lines. Owing to A549 and SPCA1 has higher level of STRIP2. Therefore, we selected A549 and SPCA1 cells conducted assays. We found that depletion of STRIP2 in H1975 cells inhibited cell proliferation and migration. Based on above findings, we proposed that STRIP2 exerts an essential function in regulating pathologic progression of LUAD.

The crucial finding of this manuscript was to identify a prognosis-related ceRNA regulatory network (TMPO-AS1/let-7c-5p/STRIP2) in LUAD. In this ceRNA regulatory network, let-7c-5p was significantly and negatively correlated with STRIP2 expression, and TMPO-AS1 was significantly and negatively correlated with let-7c-5p expression, and significantly and positively correlated with STRIP2 expression. Furthermore, TMPO-AS1 and STRIP2 were significantly increased in LUAD tissues compared to normal tissues, and survival analysis results suggested that the higher expression group had a poorer prognosis compared to the low expression group, whereas let-7c-5p exhibited lower expression in LUAD tissues compared to normal tissues, and survival analysis revealed that the lower expression group had a poorer prognosis compared to the higher expression group. These results consistently indicate that TMPO-AS1/let-7c-5p/STRIP2 is a poor prognosis-associated ceRNA regulatory network in LUAD.

This study improves our understanding of the correlation between STRIP2 and LUAD, but some limitations still exist. First, although we explored the correlation between STRIP2 and immune infiltration in LUAD patients, there is a lack of experiments to validate the function of STRIP2 in the tumor microenvironment regulation of LUAD. Second, we found that knockdown of STRIP2 inhibits cell proliferation and cell migration of LUAD. However, the potential molecular mechanisms of STRIP2 in cancer progression need to be explored in further studies.

### Conclusion

This finding describes, for the first time, the clinical relevance, immuno-oncology features and biological function of the TMPO-AS1/let-7c-5p/STRIP2 network, which upregulates STRIP2 expression in LUAD and is associated with adverse clinical outcomes. In summary, STRIP2 is a promising prognostic factor, and its future application may help determine the optimal treatment strategy for lung adenocarcinoma.

## Data Availability Statement

The original contributions presented in the study are included in the article/**Supplementary Material**. Further inquiries can be directed to the corresponding authors.

## Author Contributions

JW, YY, and LT designed this work and performed related assay. HZ and DZ analyzed the data. CL, LD, and XJ supervised and wrote the manuscript. All authors listed have made a substantial, direct, and intellectual contribution to the work and approved it for publication.

## Funding

This work was supported by National Nature Science Foundation of China (82160508) and Yunnan Applied Basic Research Projects (YNWRMY-2019-067, 2019FE001) and Yunnan Province Specialized Training Grant for High-Level Healthcare Professionals (D-201614).

## Conflict of Interest

The authors declare that the research was conducted in the absence of any commercial or financial relationships that could be construed as a potential conflict of interest.

## Publisher’s Note

All claims expressed in this article are solely those of the authors and do not necessarily represent those of their affiliated organizations, or those of the publisher, the editors and the reviewers. Any product that may be evaluated in this article, or claim that may be made by its manufacturer, is not guaranteed or endorsed by the publisher.
