# Corrigendum: Long non-coding RNA-TMPO-AS1 as ceRNA binding to let-7c-5p upregulates STRIP2 expression and predicts poor prognosis in lung adenocarcinoma

**DOI:** 10.3389/fonc.2022.1109637

**Published:** 2022-12-13

**Authors:** Juan Wang, Yixiao Yuan, Lin Tang, Haoqing Zhai, Dahang Zhang, Lincan Duan, Xiulin Jiang, Chen Li

**Affiliations:** ^1^ Department of Thoracic Surgery, The Third Affiliated Hospital of Kunming Medical University, Kunming, China; ^2^ Laboratory of Animal Models and Human Disease Mechanisms of Chinese Academy of Sciences & Yunnan Province, Kunming Institute of Zoology, Kunming, China; ^3^ Department of Biology, Chemistry, Pharmacy, Free University of Berlin, Berlin, Germany

**Keywords:** striatin-interacting protein 2, prognosis biomarkers, tumor immune infiltration, lung adenocarcinoma, cell proliferation

In the original article there was a mistake in the author affiliation for Haoqing Zhai which was erroneously presented as “Department of Thoracic Surgery, The Third Affiliated Hospital of Kunming Medical University, Kunming, China.”, it should instead be “Laboratory of Animal Models and Human Disease Mechanisms of Chinese Academy of Sciences & Yunnan Province, Kunming Institute of Zoology, Kunming, China”.

The authors apologize for this error and state that this does not change the scientific conclusions of the article in any way. The original article has been updated.

