# Supplementary material for: Long Non-Coding RNA-TMPO-AS1 as ceRNA Binding to let-7c-5p Upregulates STRIP2 Expression and Predicts Poor Prognosis in Lung Adenocarcinoma
Source: Front Oncol. 2022 Jun 14;12:921200. doi: 10.3389/fonc.2022.921200 (PMC9237420; doi:10.3389/fonc.2022.921200)
Supplement: Supplementary file 1 [file Table_1.docx]

Supplementary Material

# Supplementary Table 1 Targeted miRNAs in the ceRNA network associated with STRIP2 expression.

| hsa-let-7a-5p |
| --- |
| hsa-let-7a-5p |
| hsa-let-7b-5p |
| hsa-let-7b-5p |
| hsa-let-7c-5p |
| hsa-let-7c-5p |
| hsa-let-7d-5p |
| hsa-let-7d-5p |
| hsa-let-7e-5p |
| hsa-let-7e-5p |
| hsa-let-7f-5p |
| hsa-let-7f-5p |
| hsa-miR-15a-5p |
| hsa-miR-15a-5p |
| hsa-miR-16-5p |
| hsa-miR-16-5p |
| hsa-miR-17-5p |
| hsa-miR-20a-5p |
| hsa-miR-21-5p |
| hsa-miR-21-5p |
| hsa-miR-23a-3p |
| hsa-miR-33a-5p |
| hsa-miR-33a-5p |
| hsa-miR-93-5p |
| hsa-miR-96-5p |
| hsa-miR-98-5p |
| hsa-miR-98-5p |
| hsa-miR-101-3p |
| hsa-miR-101-3p |
| hsa-miR-103a-3p |
| hsa-miR-106a-5p |
| hsa-miR-107 |
| hsa-miR-196a-5p |
| hsa-miR-199a-5p |
| hsa-miR-199a-5p |
| hsa-miR-208a-3p |
| hsa-miR-208a-3p |
| hsa-miR-208a-3p |
| hsa-miR-129-5p |
| hsa-miR-129-5p |
| hsa-miR-147a |
| hsa-miR-147a |
| hsa-miR-34a-5p |
| hsa-miR-34a-5p |
| hsa-miR-181a-5p |
| hsa-miR-181a-5p |

Supplementary Table 2 Targeted lncRNAs in the ceRNA network associated with STRIP2 expression.

| ZNF436-AS1 |
| --- |
| SNHG12 |
| AL050341.2 |
| AL360270.2 |
| AC239868.1 |
| AC234582.1 |
| AL590666.2 |
| AL590666.2 |
| MIR29B2CHG |
| AL359924.1 |
| AC074117.1 |
| AC074117.1 |
| AC074117.1 |
| LINC01806 |
| AC105760.2 |
| LMCD1-AS1 |
| AC124045.1 |
| CCDC37-AS1 |
| AC097103.2 |
| MUC20-OT1 |
| LINC00885 |
| LINC02432 |
| AC093908.1 |
| SLC9A3-AS1 |
| LINC02242 |
| SNHG4 |
| SNHG4 |
| SNHG4 |
| CARMN |
| HEIH |
| HEIH |
| AC138035.1 |
| AL024498.1 |
| HCG18 |
| IER3-AS1 |
| AC080080.1 |
| AC004520.1 |
| HOXA11-AS |
| TRG-AS1 |
| LINC00265 |
| AC073335.2 |
| AC004921.1 |
| STAG3L5P-PVRIG2P-PILRB |
| AC023632.6 |
| CDKN2B-AS1 |
| AL590705.3 |
| LINC00963 |
| AL157392.3 |
| AL157392.3 |
| AC010997.4 |
| AL132656.4 |
| NUTM2A-AS1 |
| OLMALINC |
| RPARP-AS1 |
| LINC01001 |
| AC240565.2 |
| KCNQ1OT1 |
| KCNQ1OT1 |
| KCNQ1OT1 |
| KCNQ1OT1 |
| TMPO-AS1 |
| KCNQ1OT1 |
| LINC00294 |
